# Diagnosis of SARS-Cov-2 Infection by RT-PCR Using Specimens Other Than Naso- and Oropharyngeal Swabs: A Systematic Review and Meta-Analysis

**DOI:** 10.3390/diagnostics11020363

**Published:** 2021-02-21

**Authors:** Vânia M. Moreira, Paulo Mascarenhas, Vanessa Machado, João Botelho, José João Mendes, Nuno Taveira, M. Gabriela Almeida

**Affiliations:** 1 Área Departamental de Engenharia Química, Instituto Superior de Engenharia de Lisboa, Rua Conselheiro Emídio Navarro 1, 1959-007 Lisboa, Portugal; vania.morz@gmail.com; 2 Centro de Investigação Interdisciplinar Egas Moniz (CiiEM), Egas Moniz–Cooperativa de Ensino Superior CRL, Campus Universitário, Quinta da Granja, 2829-511 Caparica, Portugal; pmascarenhas@egasmoniz.edu.pt (P.M.); vmachado@egasmoniz.edu.pt (V.M.); jbotelho@egasmoniz.edu.pt (J.B.); jmendes@egasmoniz.edu.pt (J.J.M.); ntaveira@ff.ulisboa.pt (N.T.); 3 Evidence-Based Hub, CiiEM, Egas Moniz–Cooperativa de Ensino Superior CRL, Campus Universitário, Quinta da Granja, 2829-511 Caparica, Portugal; 4 Research Institute for Medicines, Faculty of Pharmacy, University of Lisbon, 1649-003 Lisbon, Portugal; 5 UCIBIO, REQUIMTE, Faculdade de Ciências e Tecnologia, Universidade Nova de Lisboa, 2829-516 Monte de Caparica, Portugal

**Keywords:** COVID-19, SARS-CoV-2, diagnostic, specimens, swab, saliva, deep-throat saliva, sputum, urine, feces, tears

## Abstract

The rapid and accurate testing of SARS-CoV-2 infection is still crucial to mitigate, and eventually halt, the spread of this disease. Currently, nasopharyngeal swab (NPS) and oropharyngeal swab (OPS) are the recommended standard sampling techniques, yet, these have some limitations such as the complexity of collection. Hence, several other types of specimens that are easier to obtain are being tested as alternatives to nasal/throat swabs in nucleic acid assays for SARS-CoV-2 detection. This study aims to critically appraise and compare the clinical performance of RT-PCR tests using oral saliva, deep-throat saliva/posterior oropharyngeal saliva (DTS/POS), sputum, urine, feces, and tears/conjunctival swab (CS) against standard specimens (NPS, OPS, or a combination of both). In this systematic review and meta-analysis, five databases (PubMed, Scopus, Web of Science, ClinicalTrial.gov and NIPH Clinical Trial) were searched up to the 30th of December, 2020. Case-control and cohort studies on the detection of SARS-CoV-2 were included. The methodological quality was assessed using the Quality Assessment of Diagnostic Accuracy Studies 2 (QUADAS 2). We identified 1560 entries, 33 of which (1.1%) met all required criteria and were included for the quantitative data analysis. Saliva presented the higher accuracy, 92.1% (95% CI: 70.0–98.3), with an estimated sensitivity of 83.9% (95% CI: 77.4–88.8) and specificity of 96.4% (95% CI: 89.5–98.8). DTS/POS samples had an overall accuracy of 79.7% (95% CI: 43.3–95.3), with an estimated sensitivity of 90.1% (95% CI: 83.3–96.9) and specificity of 63.1% (95% CI: 36.8–89.3). The remaining index specimens could not be adequately assessed given the lack of studies available. Our meta-analysis shows that saliva samples from the oral region provide a high sensitivity and specificity; therefore, these appear to be the best candidates for alternative specimens to NPS/OPS in SARS-CoV-2 detection, with suitable protocols for swab-free sample collection to be determined and validated in the future. The distinction between oral and extra-oral salivary samples will be crucial, since DTS/POS samples may induce a higher rate of false positives. Urine, feces, tears/CS and sputum seem unreliable for diagnosis. Saliva testing may increase testing capacity, ultimately promoting the implementation of truly deployable COVID-19 tests, which could either work at the point-of-care (e.g. hospitals, clinics) or at outbreak control spots (e.g., schools, airports, and nursing homes).

## 1. Introduction

The COVID-19 outbreak was designated a pandemic by the World Health Organization (WHO) on 11th March 2020. Since then, COVID-19 has been rapidly spreading around the globe. By the end of 2020, the number of deaths had totaled more than 1.7 million, and 80 million people had tested positive for SARS-CoV-2 worldwide, though the actual numbers are expected to be much higher [1]. One of the greatest challenges of SARS-CoV-2 is its high transmissibility rate, that drastically increases the number of infected people in a short amount of time [2,3]. A timely and reliable diagnosis is, thus, vital in preventing the spread of SARS-CoV-2, and an immense effort has been made to test as many people at risk as possible, regardless of them being symptomatic or not. On the one hand, positive test results allow physicians to promptly prescribe the correct therapy (which is particularly important when patients present co-morbidities and increased risk of severe outcomes [4]), and to isolate viral carriers, thus preventing further transmissions. On the other hand, massive testing ensures a better understanding of the disease’s progression and public health management as well as the pandemic’s epidemiology [5].

Diagnosis of SARS-CoV-2 infection can be done in three different ways. Direct diagnostic assays target the viral RNA genome (NUC assays) or a viral antigen (antigen assays), which typically is a viral surface protein. Indirect antibody assays assess the human immune response to the coronavirus infection [5,6]. The detection of viral RNA using real-time reverse-transcription polymerase chain reaction (RT-PCR) technology is the gold standard test to confirm SARS-CoV-2 infection. Specimens are collected from the upper respiratory tract (URT) such as nasopharyngeal swabs (NPS) and/or oropharyngeal swabs (OPS) since the viral load tends to be higher therein, thus improving the sensitivity and reliability of the results [6,7,8]. However, this procedure requires training and specific cautions, especially when dealing with elderly people or children [3,9], and with patients that have had recent nasal trauma or have a deviated nasal septum, among other complications [10]. In addition, it can cause discomfort to patients, and may pose a high risk of transmission, putting greater strain on both resources (such as protective equipment) and professionals [6,7,11].

The urgent demand for test kits for decentralized detection of SARS-CoV-2 infections has fueled a new frontier of diagnostic innovation. Initially, a number of miniaturized systems for nucleic acid tests based on PCR technology were used. Currently, however, new commercial in vitro diagnostic medical devices (IVDs) are being utilized in antigen testing at the point-of-care or even in laboratory settings, such as the rapid tests provided by Abbott (Panbio COVID-19 Ag), RapiGen (Biocredit COVID19), Liming Bio-Products (StrongStep COVID-19), Savant Biotechnology (Huaketai New Coronavirus), and Bioeasy Biotechnology (Diagnostic Kit for 2019-nCoV Ag Test), among others. Nevertheless, these tests are validated for URT swabs only [5].

Aiming at simplifying the sample collection procedure, so that the average person could perform self-sampling, alternative specimens have been tested for the detection of SARS-CoV-2, namely sputum, saliva, tears/conjunctival swab (CS), feces, rectal swab, urine, breast milk, and semen [12,13,14,15,16,17,18,19,20,21,22,23,24,25,26,27,28,29,30,31,32,33,34,35,36,37,38,39,40,41,42,43]. To the best of our knowledge, until now, just one protocol for saliva testing, the SalivaDirect, has been approved by a public health authority, the United States Food and Drug Administration (FDA) [44]. Still, the accuracy of saliva-based tests for clinical use remains controversial. A preliminary meta-analysis published in August 2020 revealed that the sensitivity of saliva tests is promising (91%), though it is lower than that of nasal swabs based assays (98%) [3]. The lack of data on specificity did not allow for a statistically significant analysis of this parameter and therefore, on the tests’ accuracy. Possibly, the main problem resided in the high variety and heterogeneity of studies (and results) for each specimen [3].

Considering the ever-growing number of scientific articles comparing alternative specimens for SARS-CoV-2 infection diagnosis, a more comprehensive and systematic review of the currently available literature providing meta-analytical estimates would be timely and of the utmost importance. In this way, we aim to contribute to clarify whether specimens other than the conventional nasal/throat swab specimens can be used to diagnose and manage SARS-CoV-2 infection. Therefore, we have systematically appraised and compared the overall accuracy of nucleic acid assays run with index specimens (saliva, deep-throat saliva/posterior oral samples (DTS/POS], sputum, urine, feces, and tears/CS), against standard NPS/OPS based test results. 

## 2. Materials and Methods

### 2.1. Protocol

This systematic review was submitted to PROSPERO (ID: CRD42021223894) and used the Preferred Reporting Items for Systematic Reviews and Meta-Analysis (PRISMA) guidelines [45]. The PRISMA checklist is available as a Supplemental information file (Supplementary S1, pp. 2–3).

### 2.2. Focused Question and Eligibility Criteria

The following PECO question was set: “Are physiological specimens collected without invasive swabs as accurate as the NPS/OPS specimens in the detection of SARS-CoV-2 infection by nucleic acid assays?” The outcome will include diagnostic tests accuracy estimates and also cycle thresholds (CT, number of cycles needed to amplify viral RNA to reach a detectable level), as a secondary measure of sensitivity in matched samples.

Studies were deemed eligible as per the following criteria:

- Observational studies (i.e., cross-sectional, case-control or cohort study types);
- Use of RT-PCR to detect the presence of SARS-CoV-2 in matched samples;
- Report SARS-CoV-2 positive and negative test results, and/or cycle threshold (CT) from index alternative specimens (saliva, DTS/POS, sputum, urine, feces, or tears/CS) evaluated against NPS and/or OPS;
- Studies with confirmed or suspected cases of SARS-CoV-2 infection.

Saliva samples refer to samples collected from the oral region (i.e., circumscribed to the oral cavity) while DTS/POS refers to salivary samples mixed with pharyngeal secretions. Sputum refers to primarily lower respiratory tract mucous mixed with pharyngeal and salivary secretions.

### 2.3. Search Strategy and Study Selection

Search strategies were carried out in different databases (PubMed, Scopus, Web of Science, ClinicalTrial.gov and NIPH Clinical Trial) until 30th of December 2020.

We used the following search syntax: (COVID-19 OR COVID19 OR n-CoV19 OR SARS-CoV-2 OR SARS-CoV2) AND (Diagnosis OR Diagnostic OR Test OR Detection) AND (Saliva OR Salivary OR “Oral fluid” OR Sputum OR Expectoration OR Gob OR Tears OR Conjunctival OR Stool OR Feces OR Fecal OR Urine). No restrictions on the year of publication nor on language were made. We used Mendeley reference manager (Elsevier, Mendeley Ltd, London UK) to organize records and remove duplicates. The study selection was assessed independently by two investigators (V.M.M. and P.M.), and by screening the titles and abstracts of retrieved studies. Articles selected at this point were further appraised by full text reading. Inter-examiner reliability after full-text assessment was computed through Cohen’s kappa statistics, and any disagreements were resolved by discussion with a third author (M.G.A.).

### 2.4. Data Extraction Process and Data Items

Two authors (V.M.M. and P.M.) independently retrieved and reviewed the following data (if available) from all included studies: year of publication, first author, location, design, population size, mean age, gender ratio, mean days after symptoms onset, specimens and methods used; and the following test outcomes: number of total, positives, negatives, and CTs.

### 2.5. Risk of Bias Assessment

The methodological quality of the included studies was evaluated independently by two authors (V.M.M. and P.M.), using the Quality Assessment of Diagnostic Accuracy Studies 2 (QUADAS-2) tool [46], with any discordant rating resolved by a third author (M.G.A.). This instrument judges the risk of bias (RoB) and accessibility from diagnostic accuracy studies. QUADAS-2 contains four key domains (patient selection, index test, reference standard, and flow and timing) and each domain is rated as low, high, and unclear RoB. The robvis tool was used to generate all the RoB plots [47]. If a study failed to provide enough information, the domain was classified as “no information”.

### 2.6. Quantitative Analyses

We used MetaDTA [48] to examine the overall SARS-CoV-2 detection test accuracy and perform subgroup sensitivity analysis for the selected index specimens. In MetaDTA, the bivariate random-effects model meta-analyses pooled estimates for sensitivity and specificity together. This approach accounts for potential threshold effects and covariance between sensitivity and specificity. However, because these two parameters depend on many other factors, accuracy heterogeneity is expected to be high and problematic to estimate [49]. Diagnostic odds ratios (dOR) were directly obtained from the sensitivity and specificity logit estimates. Furthermore, the summary receiver operating characteristic (sROC) plot was rendered using parameters estimated from the bivariate model through the equivalence equations of Harbord et al [50]. CTs random effects meta-analysis, and all meta-regressions to identify potential sources of heterogeneity or confounding within or between the evaluated index specimens meta-analysis were performed with OpenMeta-Analyst [51]. The influence of the specific time of sampling and the disease stage on the accuracy rate of the test were planned to be assessed through meta-regression. 

## 3. Results

**Figure 1:**
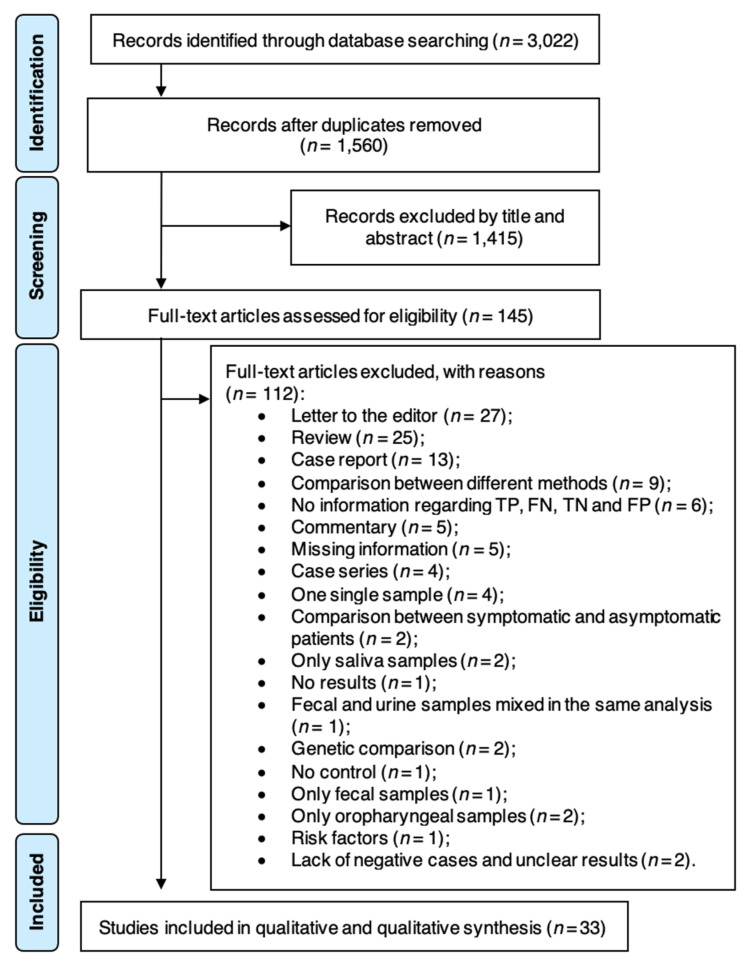
Preferred Reporting Items for Systematic Reviews and Meta-Analysis (PRISMA) flow diagram.

Electronic searches revealed a total of 3022 entries (1406 articles from PubMed, 522 from Web of Science and 1094 from Scopus). The search on clinical trial databases yielded no results. After removing replicates, 1560 articles were judged against the eligibility criteria, and 1415 were excluded after title and/or abstract review. Out of the 145 subjected to full paper review, 112 articles were excluded (Supplementary S2, pp. 4–11). As a result, a final of 33 studies met all the required criteria and were included for the quantitative data analysis (Figure 1). Inter-examiner agreement was considered as almost perfect agreement (k = 0.907, 95% CI: 0.828–0.987).

### 3.1. Characterization of the Studies

All studies utilized a PCR-based method using different targets (E, N, ORFab1, or RdRP) and compared NPS and/or OPS samples with index specimens (sputum, saliva, DTS/POS, feces, tears/CS, and urine). Twelve articles did not provide information about the control used [26,28,29,30,31,35,36,37,38,42,43,52], yet the majority used RNase P. The main characteristics of the included studies are listed in Table 1.

### 3.2. Quality Assessment

**Figure 2:**
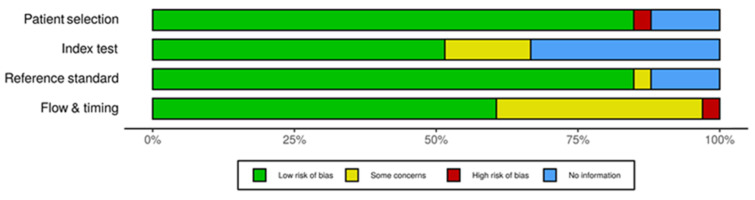
Summary of the risk of bias of the included studies (Quality Assessment of Diagnostic Accuracy Studies 2—QUADAS-2).

Overall, twenty-one studies had low risk of bias (63.6%) [12,13,14,15,16,17,18,19,20,21,22,23,24,25,35,38,39,40,41,42,43], eleven raised some concerns (33.3%) [21,26,27,28,29,30,31,32,33,34,36] and one had high risk of bias (3.0%) [37] (Figure 2) (fully detailed in Supplementary S3, pp. 12). Some studies failed to provide information regarding index tests (33.3%, *n* = 11), patient selection (12.1%, *n* = 4) and reference standard (12.1%, *n* = 4). Also, 36.4% (*n* = 12), 15.2% (*n* = 5) and 3.0% (*n* = 1) of the studies raised some concerns regarding flow and timing, index test and reference standards, respectively. Out of the total, one single study (3.0%) [37] was found to have a high risk of bias on “patient selection” and the “flow and timing” domains.

### 3.3. Quantitative Analysis (Meta-Analysis)

The random-effects meta-analysis demonstrated saliva as the index specimen with higher sensitivity and lower false-positive test results (Table 2). 

**Figure 3:**
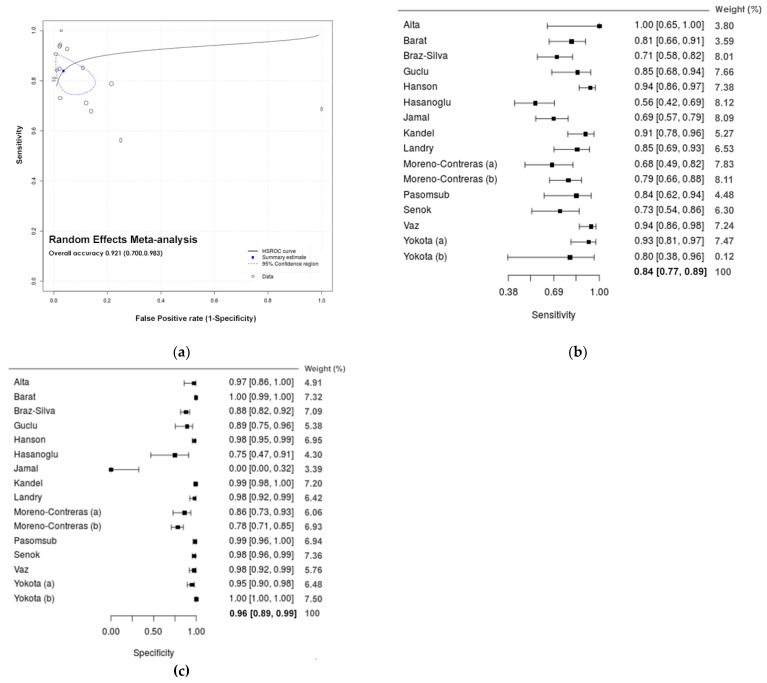
Meta-analytical estimates for saliva. (**a**) sROC plots with a curve (solid line), 95% confidence region (dashed line), summary point (blue square) (and every circle represents the sensitivity and specificity estimate from one study, and the size of the circle reflects the relative weight); (**b**) forest plot of the sensitivity; (**c**) forest plot of the specificity.

In the meta-analysis of salivary samples from the oral cavity, estimates show an overall diagnostic accuracy of 92.1% (Figure 3a; 0.921, 95% CI: 0.700; 0.983), with an estimated sensitivity of 83.9% (Figure 3b; 0.839, 95% CI: 0.774; 0.888) and specificity of 96.4% (Figure 3c; 0.964, 95% CI: 0.895; 0.988).

Meta-regressions’ screening for potential confounding variables demonstrates no influence of M/F ratio (Supplementary S4, pp. 13). Regarding the differences in the study’s sample size, while for sensitivity it is not significant (*p* = 0.518) (Figure S7), for specificity a higher sample size appears to impact positively its performance (*p* < 0.034) (Supplementary S4, pp. 13). As for the target gene, sub-analysis was deemed unsuitable given the variety of methods (Table 1).

**Figure 4:**
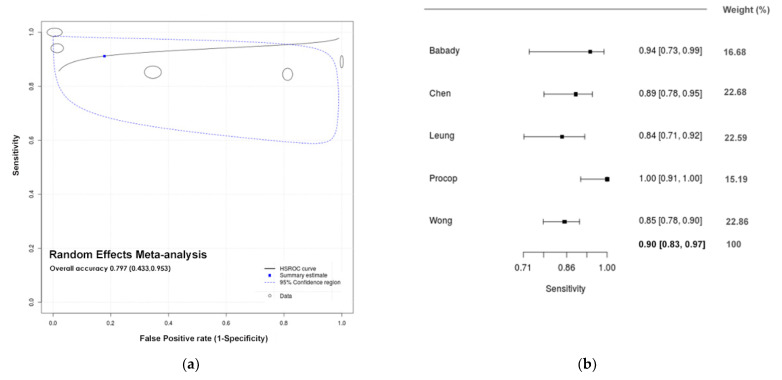

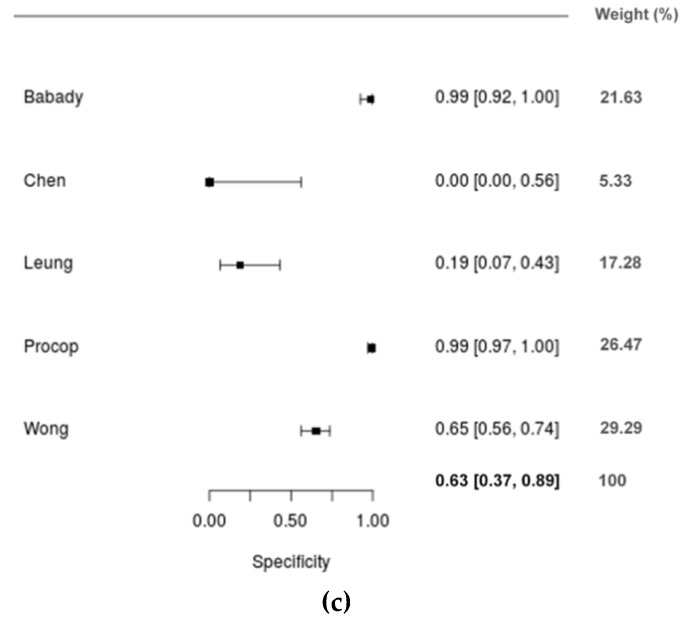
Meta-analytical estimates for DTS/POS. (**a**) summary receiver operating characteristic (sROC) plots with a curve (solid line), 95% confidence region (dashed line), summary point (blue square) (and every circle represents the sensitivity and specificity estimate from one study, and the size of the circle reflects the relative weight); (**b**) forest plot of the sensitivity; (**c**) forest plot of the specificity.

Concerning the meta-analysis of DTS/POS based tests, estimates show an overall diagnostic accuracy of 79.7% (Figure 4a; 0.797, 95% CI: 0.433; 0.953), with an estimated sensitivity of 90.1% (Figure 4b; 0.901, 95% CI: 0.833; 0.969) and specificity of 63.1% (Figure 4c; 0.631, 95% CI: 0.368; 0.893). The uncertainty of test performance estimates is much higher than in saliva-based diagnostics since less studies support the meta-analysis model fit. Meta-regression suggests that the M/F ratio have a negative confounding effect on test specificity (*p* < 0.001) (Supplementary S4, pp. 13). Estimates concerning sputum show an overall diagnostic sensitivity and specificity of 85.4% (0.875, 95% CI: 0.711; 0.952) and 25.4% (0.250, 95% CI: 0.130; 0.426), respectively. Due to the low number of studies available (*n* = 2), the sROC analysis was not performed.

Studies on tears/CS had an overall sensitivity of 17.4% (0.174, 95% CI: 0.078; 0.342) and an overall specificity of 96.1% (0.961, 95% CI: 0.127; 1.000) (Table 2) (Supplementaries S5–S6, pp. 13–14). Meta-regressions showed that specificity has a positive correlation with the M/F ratio (*p* = 0.037) (Supplementary S4, pp. 14).

In what concerns feces/anal swab, the overall diagnostic sensitivity was 46.0% (0.460, 95% CI: 0.131; 0.827) while the overall specificity was 91, 4% (0.914, 95% CI: 0.064; 0.999) (Table 2) (Supplementaries S7–S8, pp. 14–15). Meta-regressions show no confounding variables towards the performance results (Supplementary S4, pp. 13). 

Regarding urine, we did not find enough studies to compute estimates.

Finally, the CTs in RT-PCR tests were compared between the index samples under analysis. We obtained an overall mean difference between saliva and NPS/OPS of 2.792 (95% CI: −1.457; 7.041) (Supplementary S9, pp. 14), i.e., there is a negative correlation between the CT for the NPS/OPS specimen and the CT for saliva samples. This means that, on average, the CT value for saliva is higher than the one for NPS/OPS. For the mean difference between DTS/POS and NPS/OPS, a significantly different estimate was obtained: -1.808 (95% CI: −3.189;−0.427) (Supplementary S10, pp. 14).

## 4. Discussion

We systematically reviewed 33 studies on the diagnostic accuracy of RT-PCR testing using minimally invasive human specimens that may replace the nasal and throat swabbing that are routinely used for the detection of SARS-CoV-2. Overall, the most promising index specimen is saliva, with a true positive rate (sensitivity-pooled estimate) of 83.9% and a true negative rate (specificity-pooled estimate) of 96.4%. Interestingly, a critical analysis of these results shows that the accuracy of such tests was affected by a high level of heterogeneity, mostly due to methodological variations. Therefore, as a diagnostic specimen, "saliva" deserves a particular attention, and several considerations need to be taken into account. Firstly, most studies accounted for salivary samples circumscribed to the oral region (anterior to the throat) [12,13,15,20,22,26,30,33,34,35,37,38,39,40,42], while the remaining studies analyzed DTS/POS with or without pre-throat saliva [25,27,29,32,41,43]. This fact is very important as the salivary characteristics and the collection method differ, and the DTS/POS may contain samples other than the oropharyngeal region (naso-pharyngeal or laryngeal-pharyngeal) [55]. Secondly, among the studies using saliva samples from the oral cavity, the methods described show high heterogeneity and are unclear; for instance, they do not mention whether saliva was stimulated or not. Nevertheless, despite the multiple approaches used for the collection of saliva from the oral cavity (stimulated, unstimulated or unclear), saliva provided a high diagnostic accuracy (above 90%), confirming the potential of this specimen for SARS-CoV-2 detection. An additional limitation is that some of these works failed to properly describe the percentage of patients having asymptomatic, pre-symptomatic or symptomatic statuses, as the viral load varies significantly in these patients and may negatively affect the accuracy of saliva as an index specimen. To further improve the saliva collecting protocol and secure its clinical validation and utility, specifically designed studies shall be performed, to overcome the current methodological limitations.

Concerning the other evaluated index specimens, sputum presented an elevated risk of delivering false positive results when compared to NPS/OPS RT-PCR. Nonetheless, we must be cautious in interpreting these results due to the small number of studies. Similarly, tears/CS delivered the lowest sensitivity and yet, the highest specificity; though, once again, these results were based on scarce data [56].

As for the CT analyses, due to the low number of available studies, these estimates are inconclusive at this stage. 

From the sampling standpoint, both saliva and sputum can be easily obtained; however, 72% of COVID-19 patients may not produce enough sputum for analysis [57]. Therefore, saliva (from the oral region) seems to be the best specimen for both public health and epidemiologic measures [55]. Because saliva can be self-collected by patients at home or the outbreak spot, it would decrease the exposure of health-care workers to infections, and reduce the waiting times for sample collection [55]. In contrast, DTS/POS may cause the dispersion of aerosols as a result of the cough-up collection process. However, some papers have reported lower accuracy scores for salivary samples owing to critical factors such as the viral load [58], which greatly depend on the disease stage (time from onset of illness) and the time-point of specimen collection over the day. Consequently, in this systematic review we considered the influence of the specific time of sampling and the disease stage on the accuracy rate of the test through a meta-regression, though unsuccessfully. More research is needed on these factors in order to deliver more accurate results, and, eventually, to define a detailed protocol for sampling prior to collection (e.g. time-point, oral hygiene, whether to avoid drinking or eating beforehand). Other issues that may lead to false negative RT-PCR results include insufficient viral material in the specimen, laboratory error during sampling, and restrictions on sample transportation [56].

We are unaware of any other similar systematic review pooling consistent estimates on alternative specimens for detecting SARS-CoV-2, in such a way that it could have a significant impact in the accepted sampling methodologies. Indeed, almost ten months have passed since the public announcement of the COVID-19 pandemic and we now have access to a large number of scientific articles. The timing of this review is thus adequate and decisive to ensure the computation of pooling estimates, which, nonetheless, might become outdated in the months to come. Notwithstanding, these results pinpoint saliva samples circumscribed to the oral cavity as the index specimen with the greatest potential. This is a very important outcome owing to the particular circumstances we are currently experiencing (second or third waves of COVID-19) demanding extensive and rapid diagnosis of infection for which a self-administrated protocol for specimen collection would be extremely useful.

The recent understanding that some vaccines may provide little or no protection from infection with SARS-CoV2 strains bearing certain mutations in the receptor binding domain (spike variants) should prompt the development and implementation of new assays that combine sensitive diagnosis with strain identification such as those that make use of the CRISPR-Cas12 technology [59]. 

### Strengths and Limitations

Despite the thorough and comprehensive approach undertaken in this review to appraise all the clinical evidence available, some shortcomings are noteworthy. The high level of heterogeneity observed limits the validation of quantitative analyses. This might be explained by the methodological variability in different works, namely the diverse number of samples considered in each one, the fact that not all studies have used the same index test, sample treatment or target gene.

Although several studies addressed the topic of detecting the presence of SARS-CoV-2 in index samples, not all of them could be included in this meta-analysis since some of them did not provide all the raw data required to calculate the main diagnostic performance parameters. Moreover, some of the works only tested positive patients. Other factors that might have led to some variance in results are the timing of specimens’ collection and testing, sampling procedure, among others. Actually, a number of publications did not even provide such information. Given the urgency to develop effective solutions for the COVID-19 pandemic, this heterogeneity might be seen as a collateral limitation. 

These results have been derived from a rigorous protocol with up-to-date standards using appropriate guidelines. In this way we were also able to estimate the accuracy (clinical sensitivity and specificity) of a considerable number of index specimens. Still, there is an urgent need for better designed trials that should follow more homogeneous methodologies to further confirm our findings, they may aid public health authorities in validating alternative samples for SARS-CoV-2 infection diagnosis that are as reliable as nasal and throat swabs, but are non-painful, non-stressful and much easier to collect. 

## 5. Conclusions

Despite having several vaccines against SARS-CoV-2 already approved and being implemented in most developed countries, the coverage has been very slow, and it will take months to significantly reduce the prevalence of COVID-19. Since the very beginning of the pandemic, massive testing has been a critical priority in the struggle against the spread of the virus. Effective tests allow to discriminate between infected and non-infected people, thereby supporting decision making for clinical management of patients, transmission control, and epidemiological studies. According to the WHO interim guidance regarding “laboratory testing guiding principles” [59], the availability of accurate laboratory or point-of-care tests are as important as the rapid collection of appropriate physiological samples. Respiratory specimens are the only ones that were accepted up to now, but the complexity in their collection from the nasal cavity and discomfort caused to patients are driving the search for simpler and less intrusive substitutes. To this end, several alternative specimens have been compared to nasal/throat swabs for diagnosis of SARS-CoV-2 infection using nucleic acid assays (RT-PCR), and the results were systematically reviewed herein. We found that saliva from the oral region is the best candidate as an alternative specimen for SARS-CoV-2 detection. In fact, despite some heterogeneity in methodologies, the proportion of infected and non-infected patients correctly identified through the index sample is 83.9%, and 96.4%, respectively. The second-best specimen was DTS/POS, with a better true positive rate than saliva (sensitivity of 90.1%), but a much lower true negative rate (specificity of 63.1%). The specificity of sputum samples was even lower (25.4%), despite a reasonably high sensitivity (85.4%). Globally, the clinical performance of the other specimens (urine, feces, and tears) was inferior, but one should mention that the number of studies with these index specimens done so far is still scarce.

To sum up, saliva samples simply taken from the oral cavity are promising alternatives to the currently used swab-based specimens, since they can be effective, and allow self-collection. Besides mitigating the discomfort caused by sampling, saliva testing may considerably reduce the transmission risk while increasing testing capacity, ultimately promoting the implementation of truly deployable COVID-19 tests, which could either work at the point-of-care (e.g., hospitals, clinics) or outbreak control spots (e.g., schools, airports, and nursing homes). Before the index specimen saliva can be recommended by the main public health authorities, further assessment and validation is urgently required to define the best practices to adopt. 

## Figures and Tables

**Table 1:** Characteristics of the included studies.

Study	Type of Study	Date -Month (year)	Test		Information					Specimens		Main Findings	Funding
			Method (Device)	Kit (Targets)	*N*	Positive	Mean Age (Median)	Ratio M/F	Continent (Country)	Control	Index Specimen		
**Aita et al. [26]**	**Cross-sectional**	**September (2020)**	**RT-PCR (QX200 AutoDG Droplet Digital PCR System)**	One-Step RT-ddPCR Advanced Kit	43	7	63.0 (NI)	2.06	Europe (Italy)	NPS	Saliva (Stimulated)	Saliva collection can be adopted to detect SARS-CoV-2 infection in alternative to NP-swabs	NI
Babady et al. [27]	Cross-sectional	January (2021)	RT-PCR (ABI 7500 Fast, QuantStudio 5)	(N)	87	35	NI	NI	Americas (USA)	NPS	DTS/POS	Saliva is an acceptable alternative to NPSs for SARS-CoV-2 RNA detection by RT-PCR	National Cancer Institute Cancer Center (grant P30 CA008748)
Barat et al. [12]	Cohort (prospective)	December (2020)	RT-PCR (Cobas 1246800 instrument)	NucliSENS^®^easyMAG^®^platform (ORF1ab, E)	459	37	NI (42.0)	0.69	Americas (USA)	NPS/MT	Saliva (Unstimulated)	Saliva is not sensitive as NP/MT testing	National Cancer Institute, National Institutes of Health, (75N910D00024 & 75N91019F00130)
Braz-Silva et al. [13]	Cohort (prospective)	December (2020)	RT-PCR (-)	Altona RealStar^®^ SARS-CoV-2 RT-PCR Kit 1.0 (E, S)	201	22	38.3 (NI)	0.58	Americas (Brazil)	NPS	Saliva (Unstimulated)	Self-collected samples are feasible adequate alternative for SARS-CoV-2 detection	Universidade de São Paulo
Chen et al. [28]	Cross-sectional	May (2020)	RT-PCR (Xpert Xpress SARS-CoV-2 assay)	-	58	55	NI (38.0)	0.48	Asia (Hong Kong)	NPS	DTS/POS	POS and NPS were found to have similar detection rates in the point-of-care test for SARS-CoV-2 detection	Consultancy Services for Enhancing Laboratory Surveillance of Emerging Infectious Diseases and Research Capability on Antimicrobial Resistance, and Research Grants Council (T11/707/15)
Chu et al. [29]	Cohort (retrospective)	June (2020)	RT-PCR (-)	-	50	NI	NI	NI	Asia (Hong Kong)	NPS	DTS/POS	PKH pre-processing is an alternative method for nucleic acid extraction when commercial extraction kits are not available.	Public and private funding (fully disclosed in the article)
Dutescu et al. [24]	Cohort (prospective)	November (2020)	RT-PCR (Real-Time PCR Cycler iwith LightMix SarbecoV)	Superscript III one-step RT-PCR system (E)	18	13	66.3 (NI)	1	Europe (Germany)	OPS	Tears	Tear fluid and OPS lavage present a higher percentage of SARS-CoV-2	None
Güçlü et al. [37]	Cross-sectional	September (2020)	RT-PCR (-)	RT-PCR SARS-CoV-2 kit	64	30	51.0 (NI)	1.37	Europe (Turkey)	NPS/OPS	Saliva (Unclear method)	Saliva samples can be used instead of ONS samples in detecting SARS-CoV-2	NI
Hanson et al. [30]	Cohort (prospective)	October (2020)	RT-PCR (Panther Fusion system)	Hologic Aptima SARS-CoV-2 TMA (-)	354	80	35.0 (NI)	NI	Americas (USA)	NPS	Saliva (Unclear method)	Saliva is an acceptable specimen type for symptomatic patients, especially if swab or PPE 144 supplies are limited.	ARUP Institute for Clinical and Experimental Pathology
Hasanoglu et al. [35]	Cross-sectional	October (2020)	RT-PCR (-)	Bio-Speedy^®^ COVID-19 RT-qPCR Detection Kit, Bio-Rad CFX96 Touch™ (-)	60	48	33.9 (NI)	0.94	Europe (Turkey)	NPS/OPS	Saliva (Unclear method), Rectal	Asymptomatic patients have higher SARSCoV-2 viral loads than symptomatic patients. Viral load of nasopharyngeal/ oropharyngeal samples decreases with increasing disease severity.	None
Jamal et al. [38]	Cross-sectional	June (2020)	RT-PCR (-)	Allplex 2019-nCoV Assay (-)	72	64	NI (66.0)	0.85	America (Canada)	NPS	Saliva (Stimulated)	NPS were more sensitive than saliva for SARS-CoV-2 detection	Canadian Institutes of Health Research (nº. 440359) and Vanier Canada Graduate Scholarship
Kandel et al. [39]	Cohort (prospective)	November (2020)	RT-PCR (CFX96 Touch Real-time PCR detection system)	(E-gene, 5′-UTR)	429	42	NI (42.0)	NI	America (Canada)	NPS	Saliva (Stimulated)	Saliva performs comparably to NPS for the detection of SARS-CoV-2	University of Toronto
Karimi et al. [31]	Cross-sectional	May (2020)	RT-PCR (-)	NI	43	30	56.6 (NI)	2.07	Asia (Iran)	NPS	Tears	Ocular transmission of SARS-CoV-2 should be considered even in the absence of ocular manifestations	NI
Kim et al. [40]	Cross-sectional	August (2020)	RT-PCR (CFX96™ Real-time PCR detection system)	PowerChek™ 2019-nCoV Real-time PCR Kit (E, RdRP)	53	NI	NI (59.0)	NI	Asia (Korea)	NPS/OPS	Saliva (Stimulated, Sputum	Saliva is not appropriate for initial diagnosis COVID-19 to replace NP/OP swabs	Fund at the Chonnam National University (No. CNU 2020-1967).
Lai et al. [41]	Cross-sectional	August (2020)	RT-PCR (StepOnePlus Real-Time PCR System)	(N)	65	NI	NI	0.85	Asia (Hong Kong)	NPS/OPS	DTS/POS, Sputum	DTS produced the lowest viral RNA concentration and RT-PCR-positive rate compared with conventional respiratory specimens in all phases of illness	Food and Health Bureau, Hong Kong SAR Government (nº. COVID190107)
Landry et al. [42]	Cohort (prospective)	July (2020)	RT-PCR (-)	(N2)	124	33	NI	NI	Americas (USA)	NPS	Saliva (Unstimulated)	Real-time RT-PCR of pure saliva had an overall sensitivity for SARS CoV-2 RNA detection of 85.7% when compared to simultaneously collected NPS	None
Leung et al. [43]	Cohort (retrospective)	July (2020)	RT-PCR (-)	LightMix Modular SARS-CoV (COVID19)	95	45	42.0 (NI)	0.72	Asia (Hong Kong)	NPS	DTS/POS	SARS-CoV-2 detection by RT-PCR was equivalent in DTS and NPS specimens	NI
Li et al. [53]	Cross-sectional	April (2020)	RT-PCR (LightCycler 480 instrument II)	(E, N, RdRP)	12	9	52.8 (NI)	0.86	Asia (China)	NPS	Sputum, Feces	Faecal virus nucleic acid should be tested as a routine monitoring index for COVID-19	Jin hua Science and Technology Bureau (nº. 2020XG-32) and Zhejiang University special scientific research fund (nº. 2020XGZX064)
Lin et al. [23]	Cohort (retrospective)	April (2020)	RT-PCR (-)	2019-nCoV nucleic acid detection kit (E, N, ORF1ab)	52	40	57.3 (NI)	1.08	Asia (China)	TS	Sputum	The detection rates of 2019-nCoV from sputum specimens were significantly higher than those from throat swabs	Zhongnan Hospital of Wuhan University Science, Technology and Innovation Seed Fund (nº. znpy2017022)
Mesoraca et al. [14]	Cross-sectional	June (2020)	RT-PCR (iQ5 real-time PCR detection system)	Real Time Multi- plex RT-PCR kit (E, N, ORF1ab)	15	15	NI	1.29	Europe (Italy)	RT	FS	qRT-PCR assays of fecal specimens is an important step to control infection	None
Moreno-Contreras et al. [15]	Cross-sectional	September (2020)	RT-PCR (ABI Prism 7500 sequence detector system)	StarQ one-step RT-qPCR (E)	71	28	NI (41.0)	0.85	Americas (Mexico)	NPS	Saliva (Stimulated)	Saliva samples can serve as a suitable source for viral RNA detection of COVID-19	CONACyT (nº. 314343)
					182	52				NPS/OPS	Saliva (Stimulated)		
Pasomsub et al. [16]	Cross-sectional	May (2020)	RT-PCR (CFX96 Real-Time Detection System)	SARS-CoV-2 Nucleic Acid Diagnostic Kit (ORF1ab, N)	200	19	NI (36.0)	0.53	Asia (Thailand)	NPS	Saliva (Unclear method)	Saliva might be an alternative specimen for the diagnosis of COVID-19	Mahidol University
Peng et al. [17]	Cohort (retrospective)	April (2020)	RT-PCR (SLAN-96P Real-time PCR Detection System)	SARS-CoV-2 RNA Detection Kit (N)	7	NI	38.9 (NI)	NI	Asia (China)	OPS	Blood, Urine, Anal Swab	SARS-CoV-2 can infect multiple systems, including the urinary tract. Testing different specimen types may be useful for monitoring disease changes and progression, and for establishing a prognosis	National Natural Science Foundation of China (nº. 81570539, 81873572) and Guangdong Province Science and Technology Project (nº. 2020B111105001)
Perchetti et al. [18]	Cross-sectional	May (2020)	RT-PCR (ABI 7500 Real-Time PCR System)	AgPath-ID One-Step RT-PCR kit (N1, N2)	NI	NI	NI	NI	Americas (USA)	NPS	BAL, Sputum, Plasma, CSF, Stool	A modified CDC-based laboratory developed test is able to detect SARSCoV- 2 accurately with similar sensitivity across all sample types tested	University of Washington Medical Center
Procop et al. [32]	Cross-sectional	September (2020)	RT-PCR (ABI 7500 Fast Dx instruments)	(N, RdRP)	216	38	NI	0.58	Americas (USA)	NPS	DTS/POS	Saliva specimen performed as well as NPS for the qualitative detection of SARS-CoV-2 in symptomatic patients	NI
Rao et al. [19]	Cross-sectional	August (2020)	RT-PCR (-)	(E, RdRP)	217	217	NI (27.0)	NI	Asia (Malaysia)	NPS	DTS/POS	Saliva is a better alternative specimen for detection of SARS-CoV-2	National Institute of Malaysia, Ministry of Health, Malaysia (NMRR-20-860-54884)
Senok et al. [20]	Cross-sectional	August (2020)	RT-PCR (-)	NeoPlex COVID-19 kit (RdRp, N)	401	26	35.5 (NI)	4.57	Asia (United Arab Emirates)	NPS	Saliva (Unstimulated)	Saliva is a specimen with good diagnostic accuracy for SARS-CoV-2 RT-PCR	None
Sohn et al. [33]	Cross-sectional	September (2020)	RT-PCR (-)	Allplex™ 2019-nCoV Assay (E, N, RdRP)	48	48	32.6 (NI)	0.41	Asia (Korea)	NPS	Saliva (Unclear method)	Saliva can be used as a reliable specimen for the diagnosis of SARS-CoV-2 infection	None
Vaz et al. [34]	Cross-sectional	October (2020)	RT-PCR (-)	BIOMOL OneStep/ COVID-19 Kit (E, RdRP)	155	71	NI (40.0)	0.45	America (Brazil)	NPS/OPS	Saliva (Stimulated)	Use of self-collected saliva samples is an easy, convenient, and low-cost alternative to conventional NP swab-based molecular tests	NI
Wong et al. [25]	Cohort (retrospective)	June (2020)	RT-PCR (-)	LightMix^®^ Modular SARS and Wuhan CoV E-gene kit with (E)	229	122	39.0 (36.0)	NI	Asia (Hong Kong)	NPS	DTS/POS	POS is an acceptable alternative specimen to nasopharyngeal specimen for the detection of SARS-CoV-2	NI
Wu et al. [36]	Cross-sectional	March (2020)	RT-PCR (-)	-	38	28	65.8 (NI)	1.92	Asia (China)	NPS	CS	Although there is a low prevalence of SARS-CoV-2 in tears, it is possible to transmit via the eyes	National Natural Science Foundation of China (nº. 81770896 and nº. 81770920)
Yokota et al. [22]	Cross-sectional	September (2020)	RT-PCR (7500 Real-time PCR systems)	THUNDERBIRD Probe One-Step qRT-PCR kit (N2)	161	41	NI (44.9)	1.69	Asia (Japan)	NPS	Saliva (Unclear method)	Both nasopharyngeal and self-collected saliva specimens had high sensitivity and specificity	Health, Labour and Welfare Policy Research Grants 20HA2002
			RT-LAMP	Loopamp 2019-SARS-CoV-2 Detection Reagent Kit (N2)	1763	5	NI (33.5)	1.11		NPS	Saliva (Unclear method)		
Yu et al. [54]	Cross-sectional	March (2020)	RT-PCR (-)	(ORF1ab, N)	76	NI	40.0 (NI)	1	Asia (China)	NPS	Sputum	Sputum is a better indicator of viral replication in the body than throat and nasal swabs, and the viral load of sputum samples tends to increase and then decrease during the course of the disease	Beijing Ditan Hospital, Capital Medical University, and the Beijing Key Laboratory of Emerging Infectious Diseases

RT-PCR—real time PCR; NI—no information; NPS—nasopharyngeal swabs; OPS—oropharyngeal swabs; DTS/POS—nasopharyngeal swabs (NPS) and/or oropharyngeal swabs (OPS); CS—conjunctival swab; M—male; F—female; N—number of participants;

**Table 2:** Estimated diagnostic parameters for different specimens.

Specimen	*N*	Sensitivity (95% CI)	Specificity (95% CI CI)	duct (95% CI CI)	FPR (95% CI CI)	dOR (95% CI CI)
Saliva	16	0.839 (0.774;0.888)	0.964 (0.895;0.988)	2.792 (−1.457;7.041)	0.036 (0.012;0.105)	138.757 (34.059;565.290)
DTS/POS	5	0.901 (0.833;0.969)	0.631 (0.368;0.893)	−1.808 (−3.189;−0.427)	0.178 (0.014;0.763)	47.821 (1.723;1327.016)
Sputum	2	0.875 (0.711;0.952)	0.250 (0.130;0.426)	1.531 (0.301;2.762)	0.750 (0.574;0.870)	2.333 (0.624;8.719)
Tears/CS	3	0.174 (0.078;0.342)	0.961 (0.127;1.000)	−1.500 (−4.328;1.328)	0.039 (0.000;0.873)	5.155 (0.039;680.590)
Feces	3	0.460 (0.131;0.827)	0.914 (0.064;0.999)	-	0.086 (0.001;0.936)	9.016 (0.092;885.010)

CI 95% confidence interval; CS – conjunctive swab; dCT RT-PCR differential cycle threshold for reliable test in reference to NPS or OPS; FPR false positive rate; dOR diagnostic odds ratio; N – number.

## Data Availability

Available at this manuscript.

## Supplementary Materials

The following are available online at https://www.mdpi.com/2075-4418/11/2/363/s1. Supplementary S1: PRISMA 2009 Checklist, S2: Supplementary S2. List of potentially relevant studies not included in the systematic review, along with the reasons for exclusion, Supplementary S3. Methodological quality of the included studies (QUADAS-2), Supplementary S4. Meta-regressions, Supplementary S5. Subgroup analysis on tears sensitivity, Supplementary S6. Subgroup analysis on tears specificity, Supplementary S7. Subgroup analysis on feces sensitivity. Supplementary S8. Subgroup analysis on feces specificity, Supplementary S9. Forest plots of mean difference to reference sample (NPS) of CT for Saliva, Supplementary S10. Forest plots of mean difference to reference sample (NPS) of CT for DTS/POS.

## References

[1] IHME Covid Projections. https://covid19.healthdata.org/global.

[2] Budhrani A.B. (2020). A review: Coronavirus, its types, and impact of covid-19 on global wealth. Int. J. Res. Pharm. Sci..

[3] Czumbel L.M., Kiss S., Farkas N., Mandel I., Hegyi A., Nagy Á., Lohinai Z., Szakács Z., Hegyi P., Steward M.C. (2020). Saliva as a Candidate for COVID-19 Diagnostic Testing: A Meta-Analysis. Front. Med..

[4] Mitacchione G., Schiavone M., Curnis A., Arca M., Antinori S., Gasperetti A., Mascioli G., Severino P., Sabato F., Caracciolo M.M. (2020). Impact of prior statin use on clinical outcomes in COVID-19 patients: Data from tertiary referral hospitals during COVID-19 pandemic in Italy. J. Clin. Lipidol..

[5] ECDC Diagnostic Testing and Screening for SARS-CoV-2. https://www.ecdc.europa.eu/en/covid-19/latest-evidence/diagnostic-testing.

[6] Evans R.W. (2020). Diagnostic Testing for SARS-CoV-2.

[7] Ravi N., Cortade D.L., Ng E., Wang S.X. (2020). Diagnostics for SARS-CoV-2 detection: A comprehensive review of the FDA-EUA COVID-19 testing landscape. Biosens. Bioelectron..

[8] See A., Toh S.T. (2020). Respiratory sampling for severe acute respiratory syndrome coronavirus 2: An Overview. Head Neck.

[9] Sapkota D., Søland T.M., Galtung H.K., Sand L.P., Giannecchini S., To K.K.W., Mendes-Correa M.C., Giglio D., Hasséus B., Braz-Silva P.H. (2020). COVID-19 salivary signature: Diagnostic and research opportunities. J. Clin. Pathol..

[10] Marty F.M., Chen K., Verrill K.A. (2020). How to Obtain a Nasopharyngeal Swab Specimen. N. Engl. J. Med..

[11] Fernandes L.L., Pacheco V.B., Borges L., Athwal H.K., de Paula Eduardo F., Bezinelli L., Correa L., Jimenez M., Dame-Teixeira N., Lombaert I.M.A. (2020). Saliva in the Diagnosis of COVID-19: A Review and New Research Directions. J. Dent. Res..

[12] Barat B., Das S., De Giorgi V., Henderson D.K., Kopka S., Lau A.F., Miller T., Moriarty T., Palmore T.N., Sawney S. (2020). Pooled Saliva Specimens for SARS-CoV-2 Testing. J. Clin. Microbiol..

[13] Braz-Silva P.H., Mamana A.C., Romano C.M., Felix A.C., de Paula A.V., Fereira N.E., Buss L.F., Tozetto-Mendoza T.R., Caixeta R.A.V., Leal F.E. (2020). Performance of at-home self-collected saliva and nasal-oropharyngeal swabs in the surveillance of COVID-19. medRxiv.

[14] Mesoraca A., Margiotti K., Viola A., Cima A., Sparacino D., Giorlandino C. (2020). Evaluation of SARS-CoV-2 viral RNA in fecal samples. Virol. J..

[15] Moreno-Contreras J., Espinoza M.A., Sandoval-Jaime C., Cantú-Cuevas M.A., Barón-Olivares H., Ortiz-Orozco O.D., Muñoz-Rangel A.V., Hernández-De la Cruz M., Eroza-Osorio C.M., Arias C.F. (2020). Saliva sampling and its direct lysis, an excellent option to increase the number of SARS-CoV-2 diagnostic tests in settings with supply shortages. J. Clin. Microbiol..

[16] Pasomsub E., Watcharananan S.P., Boonyawat K., Janchompoo P., Wongtabtim G., Suksuwan W., Sungkanuparph S., Phuphuakrat A. (2020). Saliva sample as a non-invasive specimen for the diagnosis of coronavirus disease 2019: A cross-sectional study. Clin. Microbiol. Infect..

[17] Peng L., Liu J., Xu W., Luo Q., Chen D., Lei Z., Huang Z., Li X., Deng K., Lin B. (2020). SARS-CoV-2 can be detected in urine, blood, anal swabs, and oropharyngeal swabs specimens. J. Med. Virol..

[18] Perchetti G.A., Nalla A.K., Huang M.L., Zhu H., Wei Y., Stensland L., Loprieno M.A., Jerome K.R., Greninger A.L. (2020). Validation of SARS-CoV-2 detection across multiple specimen types. J. Clin. Virol..

[19] Rao M., Rashid F.A., Sabri F.S.A.H., Jamil N.N., Zain R., Hashim R., Amran F., Kok H.T., Samad M.A.A., Ahmad N. (2020). Comparing Nasopharyngeal Swab and Early Morning Saliva for the Identification of Severe Acute Respiratory Syndrome Coronavirus 2 (SARS-CoV-2). Clin. Infect. Dis..

[20] Senok A., Alsuwaidi H., Atrah Y., Ayedi O.A., Zahid J.A., Han A., Marzooqi A.A., Heialy S.A., Altrabulsi B., Abdelwareth L. (2020). Saliva as an alternative specimen for molecular COVID-19 testing in community settings and population-based screening. Infect. Drug Resist..

[21] Yu C., Li L., Tuersun Y., Zhao X., Feng Q., Zhang T., Tay F.R., Ma J. (2020). Oropharyngeal Secretion as Alternative for SARS-CoV-2 Detection. J. Dent. Res..

[22] Yokota I., Shane P.Y., Okada K., Unoki Y., Yang Y., Inao T., Sakamaki K., Iwasaki S., Hayasaka K., Sugita J. (2020). Mass screening of asymptomatic persons for SARS-CoV-2 using saliva. Clin. Infect. Dis..

[23] Lin C., Lin C., Xiang J., Yan M., Li H., Huang S., Huang S., Shen C., Shen C. (2020). Comparison of throat swabs and sputum specimens for viral nucleic acid detection in 52 cases of novel coronavirus (SARS-Cov-2)-infected pneumonia (COVID-19). Clin. Chem. Lab. Med..

[24] Dutescu R.M., Banasik P., Schildgen O., Schrage N., Uthoff D. (2021). Detection of Coronavirus in Tear Samples of Hospitalized Patients With Confirmed SARS-CoV-2 From Oropharyngeal Swabs. Cornea.

[25] Wong S.C.Y., Tse H., Siu H.K., Kwong T.S., Chu M.Y., Yau F.Y.S., Cheung I.Y.Y., Tse C.W.S., Poon K.C., Cheung K.C. (2020). Posterior Oropharyngeal Saliva for the Detection of Severe Acute Respiratory Syndrome Coronavirus 2 (SARS-CoV-2). Clin. Infect. Dis..

[26] Aita A., Basso D., Cattelan A.M., Fioretto P., Navaglia F., Barbaro F., Stoppa A., Coccorullo E., Farella A., Socal A. (2020). SARS-CoV-2 identification and IgA antibodies in saliva: One sample two tests approach for diagnosis. Clin. Chim. Acta.

[27] Babady N.E., McMillen T., Jani K., Viale A., Robilotti E.V., Aslam A., Diver M., Sokoli D., Mason G., Shah M.K. (2020). Performance of Severe Acute Respiratory Syndrome Coronavirus 2 Real-Time RT-PCR Tests on Oral Rinses and Saliva Samples. J. Mol. Diagn..

[28] Chen J.H.K., Yip C.C.Y., Poon R.W.S., Chan K.H., Cheng V.C.C., Hung I.F.N., Chan J.F.W., Yuen K.Y., To K.K.W. (2020). Evaluating the use of posterior oropharyngeal saliva in a point-of-care assay for the detection of SARS-CoV-2. Emerg. Microbes Infect..

[29] Chu A.W.H., Chan W.M., Ip J.D., Yip C.C.Y., Chan J.F.W., Yuen K.Y., To K.K.W. (2020). Evaluation of simple nucleic acid extraction methods for the detection of SARS-CoV-2 in nasopharyngeal and saliva specimens during global shortage of extraction kits. J. Clin. Virol..

[30] Hanson K.E., Barker A.P., Hillyard D.R., Gilmore N., Barrett J.W., Orlandi R.R., Shakirb S.M. (2020). Self-collected anterior nasal and saliva specimens versus health care worker-collected nasopharyngeal swabs for the molecular detection of SARS-CoV-2. J. Clin. Microbiol..

[31] Karimi S., Arabi A., Shahraki T., Safi S. (2020). Detection of severe acute respiratory syndrome Coronavirus-2 in the tears of patients with Coronavirus disease 2019. Eye.

[32] Procop G.W., Shrestha N.K., Vogel S., van Sickle K., Harrington S., Rhoads D.D., Rubin B.P., Terpeluk P. (2020). A Direct Comparison of Enhanced Saliva to Nasopharyngeal Swab for the Detection of SARS-CoV-2 in Symptomatic Patients. J. Clin. Microbiol..

[33] Sohn Y., Jeong S.J., Chung W.S., Hyun J.H., Baek Y.J., Cho Y., Kim J.H., Ahn J.Y., Choi J.Y., Yeom J.-S. (2020). Assessing Viral Shedding and Infectivity of Asymptomatic or Mildly Symptomatic Patients with COVID-19 in a Later Phase. J. Clin. Med..

[34] Vaz S.N., Santana D.S.D., Netto E.M., Pedroso C., Wang W.K., Santos F.D.A., Brites C. (2020). Saliva is a reliable, non-invasive specimen for SARS-CoV-2 detection. Braz. J. Infect. Dis..

[35] Hasanoglu I., Korukluoglu G., Asilturk D., Cosgun Y., Kalem A.K., Altas A.B., Kayaaslan B., Eser F., Kuzucu E.A., Guner R. (2020). Higher viral loads in asymptomatic COVID-19 patients might be the invisible part of the iceberg. Infection.

[36] Wu P., Duan F., Luo C., Liu Q., Qu X., Liang L., Wu K. (2020). Characteristics of Ocular Findings of Patients with Coronavirus Disease 2019 (COVID-19) in Hubei Province, China. Jama Ophthalmol..

[37] Güçlü E., Koroglu M., Yürümez Y., Toptan H., Kose E., Güneysu F., Karabay O. (2020). Comparison of saliva and oro-nasopharyngeal swab sample in the molecular diagnosis of COVID-19. Rev. Assoc. Med. Bras..

[38] Jamal A.J., Mozafarihashjin M., Coomes E., Powis J., Li A.X., Paterson A., Anceva-Sami S., Barati S., Crowl G., Faheem A. (2020). Sensitivity of Nasopharyngeal Swabs and Saliva for the Detection of Severe Acute Respiratory Syndrome Coronavirus 2. Clin. Infect. Dis..

[39] Kandel C., Zheng J., McCready J., Serbanescu M.A., Racher H., Desaulnier M., Powis J.E., Vojdani K., Finlay L., Sheldrake E. (2020). Detection of SARS-CoV-2 from Saliva as Compared to Nasopharyngeal Swabs in Outpatients. Viruses.

[40] Kim S.E., Lee J.Y., Lee A., Kim S., Park K.H., Jung S.I., Kang S.J., Oh T.H., Kim U.J., Lee S.Y. (2020). Viral Load Kinetics of SARS-CoV-2 Infection in Saliva in Korean Patients: A Prospective Multi-center Comparative Study. J. Korean Med. Sci..

[41] Lai C.K.C., Chen Z., Lui G., Ling L., Li T., Wong M.C.S., Ng R.W.Y., Tso E.Y.K., Ho T., Fung K.S.C. (2020). Prospective Study Comparing Deep Throat Saliva With Other Respiratory Tract Specimens in the Diagnosis of Novel Coronavirus Disease 2019. J. Infect. Dis..

[42] Landry M.L., Criscuolo J., Peaper D.R. (2020). Challenges in use of saliva for detection of SARS CoV-2 RNA in symptomatic outpatients. J. Clin. Virol..

[43] Leung E.C.M., Chow V.C.Y., Lee M.K.P., Lai R.W.M. (2020). Deep throat saliva as an alternative diagnostic specimen type for the detection of SARS-CoV-2. J. Med. Virol..

[44] FDA Emergency Use Authorization Issued in August 2020. https://www.fda.gov/news-events/press-announcements/coronavirus-covid-19-update-fda-issues-emergency-use-authorization-yale-school-public-health.

[45] Moher D., Liberati A., Tetzlaff J., Altman D.G., Altman D., Antes G., Atkins D., Barbour V., Barrowman N., Berlin J.A. (2009). Preferred reporting items for systematic reviews and meta-analyses: The PRISMA statement. Plos Med..

[46] Bristol M.S. (2014). QUADAS2: Background Document. https://www.bristol.ac.uk/media-library/sites/quadas/migrated/documents/background-doc.pdf.

[47] McGuinness L.A., Higgins J.P.T. (2021). Risk-of-bias VISualization (robvis): An R package and Shiny web app for visualizing risk-of-bias assessments. Res. Synth. Methods.

[48] Freeman S.C., Kerby C.R., Patel A., Cooper N.J., Quinn T., Sutton A.J. (2019). Development of an interactive web-based tool to conduct and interrogate meta-analysis of diagnostic test accuracy studies: MetaDTA. Bmc Med. Res. Methodol..

[49] Leeflang M.M.G. (2014). Systematic reviews and meta-analyses of diagnostic test accuracy. Clin. Microbiol. Infect..

[50] Harbord R.M., Deeks J.J., Egger M., Whiting P., Sterne J.A.C. (2007). A unification of models for meta-analysis of diagnostic accuracy studies. Biostatistics.

[51] Wallace B.C., Dahabreh I.J., Trikalinos T.A., Lau J., Trow P., Schmid C.H. (2012). End-Users: R as a Computational Back-End. J. Stat. Softw..

[52] Jeong H.W., Kim S.M., Kim H.S., Kim Y.I., Kim J.H., Cho J.Y., Kim S.H., Kang H., Kim S.G., Park S.J. (2020). Viable SARS-CoV-2 in various specimens from COVID-19 patients. Clin. Microbiol. Infect..

[53] Li Y., Hu Y., Yu Y., Zhang X., Li B., Wu J., Li J., Wu Y., Xia X., Tang H. (2020). Positive result of Sars-Cov-2 in faeces and sputum from discharged patients with COVID-19 in Yiwu, China. J. Med. Virol..

[54] Yu X., Sun S., Shi Y., Wang H., Zhao R., Sheng J. (2020). SARS-CoV-2 viral load in sputum correlates with risk of COVID-19 progression. Crit. Care.

[55] Ceron J., Lamy E., Martinez-Subiela S., Lopez-Jornet P., Capela-Silva F., Eckersall P., Tvarijonaviciute A. (2020). Use of Saliva for Diagnosis and Monitoring the SARS-CoV-2: A General Perspective. J. Clin. Med..

[56] Li Y., Yao L., Li J., Chen L., Song Y., Cai Z., Yang C. (2020). Stability issues of RT-PCR testing of SARS-CoV-2 for hospitalized patients clinically diagnosed with COVID-19. J. Med. Virol..

[57] Huang C., Wang Y., Li X., Ren L., Zhao J., Hu Y. (2020). Clinical features of patients infected with 2019 novel coronavirus in Wuhan, China. Lancet.

[58] Yoon J.G., Yoon J., Song J.Y., Yoon S.Y., Lim C.S., Seong H., Noh J.Y., Cheong H.J., Kim W.J. (2020). Clinical significance of a high SARS-CoV-2 viral load in the Saliva. J. Korean Med. Sci..

[59] Broughton J.P., Deng X., Yu G., Fasching C.L., Servellita V., Singh J., Miao X., Streithorst J.A., Granados A., Sotomayor-Gonzalez A. (2020). CRISPR–Cas12-based detection of SARS-CoV-2. Nat. Biotechnol..

